# The Impact of Protective Gloves on Manual Dexterity in Cold Environments—A Pilot Study

**DOI:** 10.3390/ijerph19031637

**Published:** 2022-01-31

**Authors:** Joanna Orysiak, Magdalena Młynarczyk, Emilia Irzmańska

**Affiliations:** 1 Department of Ergonomics, Central Institute for Labour Protection—National Research Institute, Czerniakowska St. 16, 00-701 Warsaw, Poland; m.mlynarczyk@ciop.pl; 2 Department of Personal Protective Equipment, Central Institute for Labour Protection—National Research Institute, Wierzbowa St. 48, 90-133 Lodz, Poland; emirz@ciop.lodz.pl

**Keywords:** cold environment, manual dexterity performance, hand performance, protective gloves, performance time

## Abstract

Our research aimed to determine the impact of two types of protective gloves. The research tested the glove performance on men exposed to a range of temperatures reflecting the working conditions in fruit and vegetable processing. The gloves were assessed for performance within the time required to complete a specific manual task and for performance relative to the subjective thermal sensations in the male subjects. Six males participated in a total of 3 study variants: at +5 °C (with double gloves and single glove), at −1 °C (with double gloves and single glove) and in reference conditions +20 °C (without gloves), in which they performed manual tasks. The measurement of manual task performance time was used to assess manual dexterity. Subjective thermal sensations were determined. Differences in the time required to complete specific tasks were observed between the variants with gloves (both at a temperature of +5 °C and −1 °C), and without gloves (*p* < 0.05). The type of protective gloves had an impact on the time needed to complete manual tasks and therefore may affect manual dexterity.

## 1. Introduction

Workers from various industries are exposed to different microclimate conditions—cold, hot or moderate. According to European Standard EN ISO 11079, the cold environment is defined as the thermal environment conditions for which, inter alia, the air temperature is below 10 °C [1]. There are many occupations, outdoor and indoor, where individuals are exposed to the cold, e.g., divers, electrical utility line workers, arctic surface miners, food processing workers, search and rescue, mountaineers, fisherman, and construction workers [2,3]. According to Statistics Poland data [4], in 2020 over 10,000 workers in Poland were exposed to work in a cold microclimate, including around 40% of people working in the production of food and beverages. Working in cold conditions may impact health, safety and occupational performance [3]. Cold exposure decreased manual performance due to impairment of finger mobility, grip strength, tactile sensitivity, muscle function and proprioception as well as by causing pain, increasing arousal levels and distracting attention [3]. Many studies showed that cold exposure affects the dexterity of the fingers more negatively than the dexterity of the hands [3,5]. When people work at low temperatures or in contact with cold surfaces, it is very important to ensure that the appropriate skin temperatures for hands and fingers are maintained in order to prevent a reduction in the manual performance and/or frostbite [6,7]. Workers should be equipped with appropriate gloves to protect them against the effects of low temperatures [6]. It was shown, that gloves may reduce hand heat losses even by 60–90% [8]. Moreover, gloves also protect hands from injury [3,9]. However, despite the protection and safety, wearing gloves may impair manual performance, for example, due to decreased grip strength, dexterity and tactile sensitivity [3,10,11,12,13]. For this reason, some cold room workers experienced cold fingers yet did not wear appropriate gloves, based on the belief that wearing gloves reduces manual dexterity [7,14]. This also occurs in other professions, such as diving, where divers believe that gloves are so cumbersome in some operating environments that they remove them to perform tasks that require good tactile sensitivity or precise motor control [7,13,15]. This is done even though the removal of gloves accelerates hand cooling and may lead to further deterioration of dexterity [7,13]. Therefore, gloves used by workers in cold environments should have properties allowing them to fulfill not only protective but also ergonomic functions necessary to maintain manual dexterity in such conditions [16]. Protective gloves should [6,7,16]:

- have an appropriate level of thermal insulation,
- be made of flexible materials that are not damaged by low temperature,
- be comfortable to use,
- allow workers to manipulate their fingers, and
- be fitted to the user’s hand.

Given the different working conditions and exposure to cold, correlating the properties of gloves assessed in the laboratory with the actual protection they provide to workers is difficult to assess [7,16,17]; therefore, the current protective clothing at an exposure to cold is not always sufficient to maintain manual dexterity as well as thermal balance (heat balance) [7,13,18]. For correct selection of gloves according to working conditions, it is important to know the exposure conditions, the temperature range inside the glove, the nature of the objects with which the hands of employees come into contact and the type of task performed [7,16]. Depending on the type of exposure to cold as well as the time and temperature of exposure, the cooling of employee hands may occur with different intensity and speed. Therefore, it becomes justified to use different types of protective gloves in different conditions of exposure to cold [7,16].

The impact of cold on manual dexterity is widely described in the literature. However, the effect of using gloves in this condition is not well recognized, including the influence of different designs and materials on manual dexterity in cold environment. Therefore, this knowledge should be further explored and expanded. Moreover, there are no studies on contact cold in the context of different glove applications.

The aim of our study was to determine the impact of two different type of gloves on the time required to complete a manual task and on the subjective thermal sensations in men exposed to a range of temperatures reflecting the working conditions in fruit and vegetable processing.

The authors would like to broaden knowledge of the effect of such temperatures on manual performance and thermal subjective responses when using different types of gloves. Additionally, the authors would like to check whether relying on the subjective feelings of workers was reflected in the time in which they performed the tasks.

## 2. Materials and Methods

### 2.1. Subjects

The research was conducted with men selected on the basis of the results of qualifying tests—performance tests, from which V_O2peak_ (oxygen uptake at 75% of the age-predicted maximum heart rate) was determined. Those tests were preceded by a medical interview.

Six male subjects (20.5 ± 1.9 year; 77.4 ± 9.7 kg; 1.81 ± 0.1 m; V_O2peak_: 31.0 ± 3.1 mL/min/kg), not exposed to cold environment for several weeks, participated in the study. The study included participants, who achieved the highest and similar V_O2peak_ values during qualifying tests and were healthy on the test day. The doctor carried out an examination and medical interview of all volunteers before each variant of study.

The study procedures were approved by the Committee of Ethics and all participants provided written informed consent.

### 2.2. Clothing

**Figure 1:**
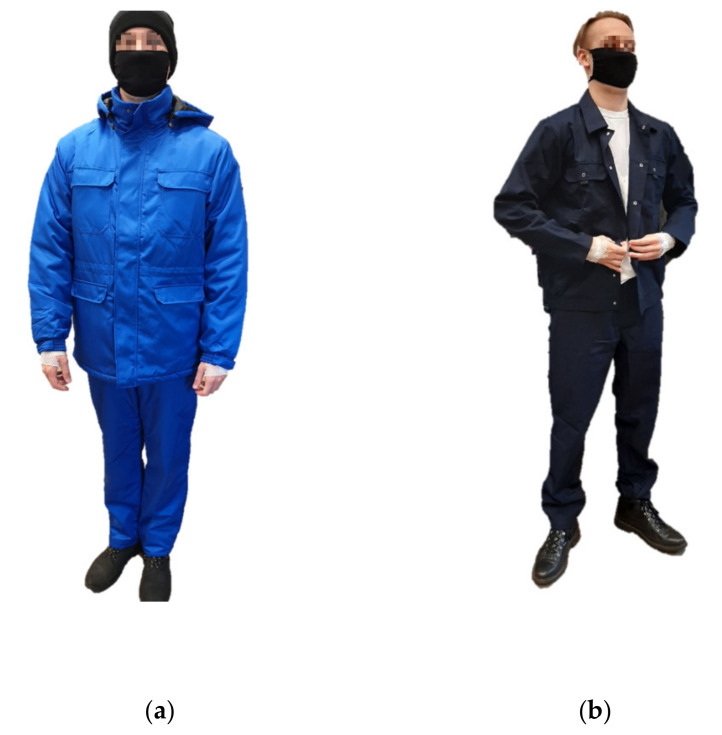
Photo of the clothing ensemble used in tests (from the left): (**a**) protective clothing for cold environment and (**b**) standard work clothing.

In order to determine the typical clothing of a worker in the fruit and vegetable industry, consultations with an occupational health and safety representative, as well as, a dress survey was carried out in one of the fruit and vegetable processing companies [19]. On the basis of the obtained results, a set of clothes for experiments was completed. The test clothing set (protective clothing for cold environment) consisted of: thermal underwear, a cotton T-shirt, a fleece jacket, woolen socks, a woolen cap, an insulated jacket, and insulated dungarees (Figure 1a). In the reference condition, subjects were dressed in standard work clothes, a cotton T-shirt and thin socks (Figure 1b). The subjects had shoes adapted to the prevailing conditions.

Additionally, two types of gloves were used during the tests: glove A—double glove (white knitwear + black fleece) and glove B—single glove (polyester). After consultations with an occupational health and safety representative, test gloves were selected, which were used by workers in the cold storage of the fruit and vegetable processing companies. Glove size was selected by volunteers before starting the study [20].

The tested protective gloves differed in the type of material and the type of fibres used. Details of glove construction were given in Table 1.

### 2.3. Research Equipment

#### 2.3.1. Climatic Chamber

Air temperature in the climate chamber (Weiss, Germany) can be simulated from −40 to +70 °C. The construction of the chamber allows control of parameters such as air velocity and relative humidity [21].

#### 2.3.2. Manual Dexterity Determination

**Figure 2:**
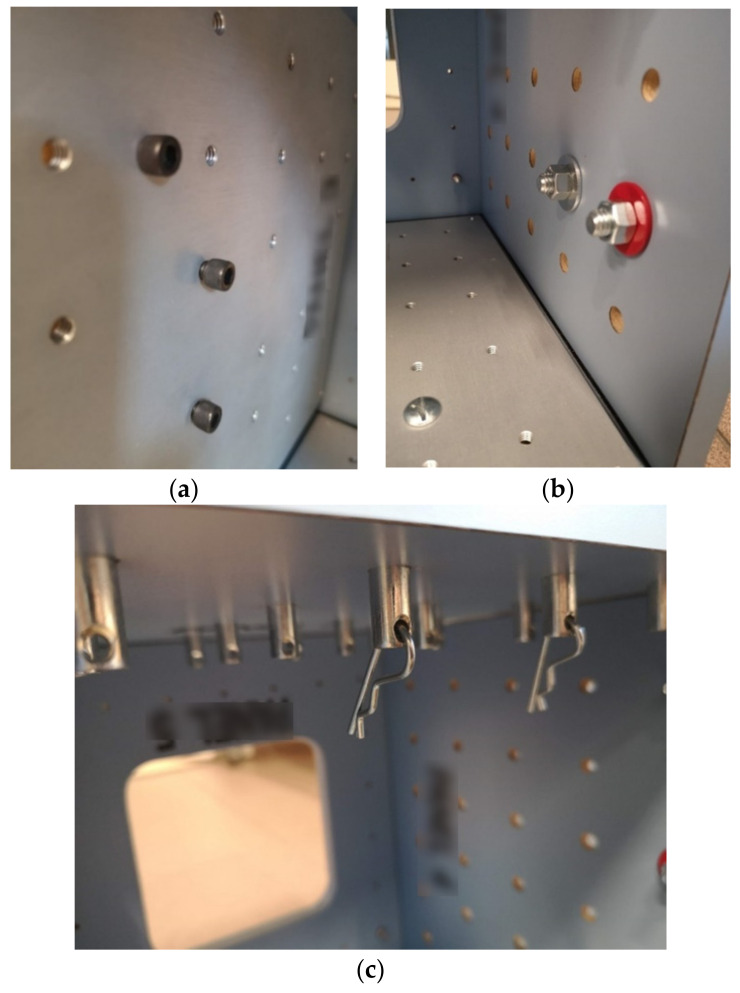
Solution example (from the left) for: (**a**) wall_1, (**b**) wall_2 and (**c**) wall_3.

Manual dexterity is defined as motor skill determined by arm, hand and finger range of motion as well as possibility of hand and fingers to manipulate [22,23]. Manual dexterity was determined using Valpar Component Work Samples 1 (VCWS 1 Small Tools (Mechanical) (Bases of Virginia, LLC, VA, USA). It allowed the determination of the ability for finger and hand precise movements and to work with small tools in tight or awkward spaces [24]. For this reason, VCWS 1 was used because it represents the general dexterity of both the fingers and the hand, but it was modified for the purposes of this study. The VCWS consisted of 5 walls, but in this experiment three walls were selected. Wall_1 consisted of screwing in small screws (in any position), wall_2 consisted of completing (screw, cap, cap, nut) and screwing the set in the two lower rows of holes on the wall, and wall_3 consisted of threading cotter pin through the holes in the bolts (Figure 2).

### 2.4. Methodology

#### 2.4.1. Qualifying Tests

Body mass and height. Body height was assessed using a personal weight C315.60/150.OW-3 (RADWAG, Poland) with an accuracy of 0.1 cm. A body composition analyzer (Inbody 270, South Korea) was used to measure the body mass.

Aerobic capacity. As part of the qualification tests, participants performed a graded exercise test to exhaustion on a cycloergometer (Monark, Sweden) to determine aerobic capacity (oxygen uptake at 75% of the age-predicted maximum heart rate—V_O2peak_). This aerobic test was preceded by temperature measurement, SARS-CoV-2 declaration and then a medical interview with resting electrocardiography. The exercise test started at workload 50W, and was increased every 3 min by 25 W. After reaching 75% of the maximum heart rate the aerobic test was terminated. An open circuit breath-by-breath automated gas analysis system (MetaLyzer system CPET, Cortex biophysik GmbH, Leipzig, Germany) was used to measure oxygen uptake. V_O2peak_ was determined as the highest value of oxygen uptake registered within 60 s during the test. On the basis of qualifying tests—the values of the oxygen uptake at 75% of the age-predicted maximum heart rate, volunteers were selected for further tests in the climatic chamber.

#### 2.4.2. Testing the Properties of Gloves and Clothing Used in the Tests

##### The Gloves

The convective cold test was carried out on a thermal hand model according to EN 511 standard [25]. Thermal insulation is defined as the resistance to dry heat loss of the hand, including the resistance from the protective glove and the air layer between the hand model and the applied glove.

According to EN 511 standard [25], the surface temperature of the thermal hand model was set at 33 °C. The parameters in the climatic chamber were air temperature 10 °C and relative humidity 50%. After stabilizing the parameters of heat exchange between air in the chamber and hand model, the convective cold (thermal insulation) was determined.

The thermal insulation of gloves, which determines their thermal effectiveness, was determined from the Equation (1):

I_TR_ = (t_hand_model_ − t_a_) × Q_hand_ (1)

where: I_TR_—resistance to dry heat loss from the hand, which includes the resistance provided by the handwear and the air layer around the dressed model; t_hand_model_—mean surface temperature of the measuring zone of the hand [°C]; t_a_—mean air temperature in the climatic chamber [°C]; Q_hand_—measured power supply to the hand measuring zone during steady state [W/m^2^].

The contact cold test was carried out on a thermal hand model with pressure simulation according to EN 511 standard [20]. Surface temperature of the thermal hand model was set at 33 °C. The parameters in the climatic chamber were air temperature 10 °C and relative humidity 50%. A pressure-simulating tester made of metal was placed on the tested gloves, with clamping by metal ball causing pressure of 6.9 kPa.

During steady state, the value of contact cold (thermal resistance in contact with a metal object) was determined.

##### The Protective Clothing for Cold Environment

The thermal insulation of the clothing ensemble was tested in the climatic chamber on the thermal manikin (NEWTON, MTNW, USA). According to the EN 342 [26] and EN ISO 15831 [27] standards, total thermal insulation was carried out under static conditions. The manikin’s surface was set on 34 °C, and the microclimate parameters in the chamber were: air temperature 2.4 °C, relative humidity 38% and air velocity ~0.4 m/s.

The total thermal insulation was calculated from the Equation (2) [27,28,29]:

I_t_ = (t_s_ − t_a_) × A/H_c_, (2)

where: It—total thermal insulation [m2°C/W]; ts—mean manikin’s surface temperature [°C] ta—air temperature in climatic chamber [°C]; A—total surface area of manikin [m2]; Hc—total heat loss [W].

#### 2.4.3. Volunteer Studies

**Figure 3:**
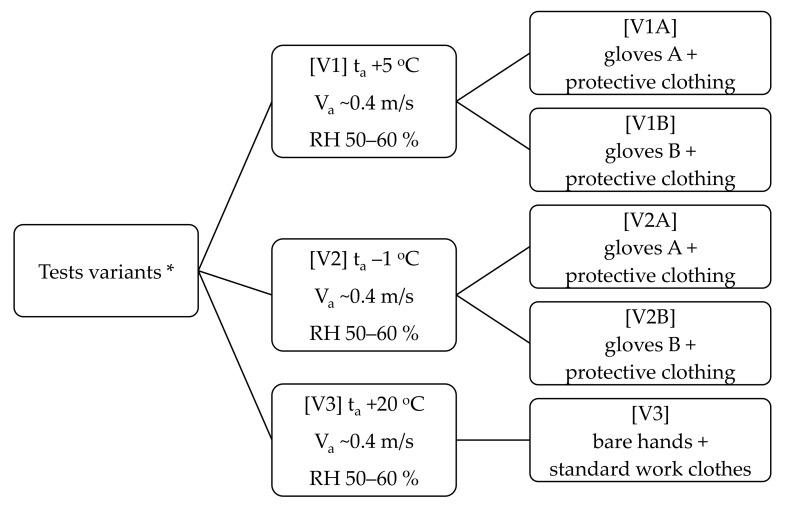
Detailed description of the test variants (* thermal condition monitor by microclimate meter (INNOVA 1221, LumaSense Technologies A/S, Denmark); t_a_—air temperature, V_a_—air velocity, RH—relative humidity).

Each participant underwent 3 study variants: at +5 °C (gloves A and B), at −1 °C (gloves A and B) and +20 °C (bare hands, control conditions). Temperature values were based on consultations with an occupational health and safety representative of the fruit and vegetable processing companies in Poland. The control conditions were to ensure the greatest possible comfort in the performance of individual tasks, which was why no gloves were used and work clothes appropriate to the air temperature work were used (Figure 3).

Participants spent 45 min in the climatic chamber for each study variant. During this time, they performed as follows:

- Walking (2.5 km/h) on the treadmill (simulated moderate effort)—5 min;
- Sorting of small elements by shape;
- Completing the wall 1 (time measurement)—1st repetition;
- Completing the wall 2 (time measurement)—1st repetition;
- Completing the wall 3 (time measurement)—1st repetition;
- Sorting of small elements by color;
- Sorting of small elements by shape;
- Completing the wall 1 (time measurement)—2nd repetition;
- Completing the wall 2 (time measurement)—2nd repetition;
- Completing the wall 3 (time measurement)—2nd repetition.

**Figure 4:**
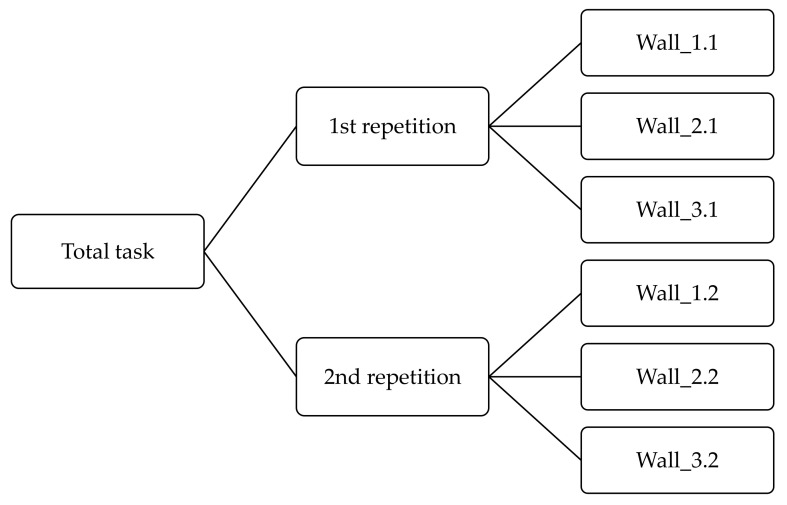
Scheme of total task performed by participants.

In our study, the time needed to complete: individual walls in 1st repetition and in 2nd repetition as well as the total time to complete all walls was considered as a measure of manual dexterity [23,30] (Figure 4).

The order of manual tasks was the same under all study variants. The VCWS was placed on the table at a height of ca. 1 m. Participants using only hands, in a standing position, completed as follows: wall_1, wall_2 and wall_3. The filling of the three walls was equivalent to performing one repetition (Figure 4). The total task consisted of performing two repetitions (1st repetition and 2nd repetition). To reduce the learning effect, participants were familiar with the procedure for filling the walls and the order of the gloves (A or B) was assigned randomly to each participant [31].

The researchers recorded by stopwatch the time required to complete the tasks during the experiment. After completing all three walls, the participants answered questions about subjective sensation. Participants were asked to discuss the effect of gloves on manual dexterity, comfort of using the gloves and thermal comfort for hands [32]. The subjective scale of thermal comfort, ease of manipulation and comfort of use were presented in Table 2.

#### 2.4.4. Statistical Analysis

Friedman’s ANOVA was used to examine differences between variants in the experiment, followed by post-hoc comparisons if necessary. The level of statistical significance was set at *p* < 0.05.

## 3. Results

### 3.1. Tested Clothing Parametres

#### 3.1.1. Clothing Insulation

The total thermal insulation of protective clothing was 0.415 m^2^ °C/W (2.68 clo). According to the EN 342 [26], the clothing ensemble can be used during light physical activity (8 h of work time) at an air temperature of 0 °C and during moderate physical activity (8 h) at −16 °C. According to the EN ISO 11079 standard [1], the selected clothing ensemble can be used for up to 8 h both in a cold environment at an air temperature from −20 to 10 °C, in the case of moderate work (average physical activity 170 W/m^2^), as well as from −5 to 10 °C, in the case of light work (workers perform light physical activity). It follows that the clothing ensemble can be used during works at an air temperature of both +5 °C and −1 °C in our study condition.

#### 3.1.2. Glove Parameters

Convective cold tests of gloves have shown that, in terms of thermal insulation, the gloves have comparable properties (Table 3).

Tested gloves achieved the lowest level (1st performance level) of resistance to convective cold.

The results of contact cold tests of gloves have shown a difference in terms of thermal resistance (Table 4).

Tested gloves achieved a medium level (2nd and 3rd performance level) of resistance to convective cold.

### 3.2. Time to Complete the Tasks

#### 3.2.1. Time to Complete the Total Tasks

The time to complete the total task, or one repetition, as well as the time required to complete the individual wall as part of 1st and 2nd repetitions in a specific test variant were analyzed. The time needed to complete two repetitions and separately 1st and 2nd repetitions were presented in Table 5, and the obtained results were compared with reference values (values obtained in variant V3).

Significant differences were observed between time to complete the total task (*p* = 0.014), 1st repetition (*p* = 0.006) and 2nd repetition (*p* = 0.012). Both in the case of the task performance time of two repetitions and of individual repetition, separately, variants V1A, V1B, V2A and V2B differed from variant V3 (for two repetitions *p* = 0.028, for 1st repetition *p* = 0.028 and for 2nd repetition *p* = 0.028) (Table 5).

Only in variant V1A differences were observed between times for the 1st and 2nd repetition (p = 0.028). The volunteers took longer to complete the 1st repetition than the second (00:12:59 and 00:11:14, respectively).

#### 3.2.2. Time to Complete the Individual Walls

The performance time of individual walls was summarized in Table 6, and the obtained results were compared with reference values (values obtained in variant V3). Differences between the tested variants were observed for walls in both repetitions (wall 1.1 *p* = 0.012; wall 1.2 *p* = 0.005; wall 2.1 *p* = 0.012; wall 2.2 *p* = 0.015; wall 3.1 *p* = 0.007 and wall 3.2 *p* = 0.001). The significant differences between variant V3 and other variants were presented in Table 6.

Significant differences were observed between V1A wall 3.1 and V2A wall 3.1 (*p* = 0.046); V1A wall 3.2 and V2A wall 3.2 (*p* = 0.028), referring to the impact of temperature; and V2A 3.2 and V2B 3.2 (*p* = 0.028), referring to the impact of type of gloves. Moreover, when the same thermal conditions and type of gloves were compared, it was observed that volunteers completed the 1st repetition of wall 2 in longer time than for the 2nd repetition of wall 2 at +5 °C using gloves B (V1B wall 2.1 vs V1B wall 2.2 *p* = 0.028).

### 3.3. Thermal Comfort Scale, Ease of Manipulation Response and Comfort of Use

**Figure 5:**
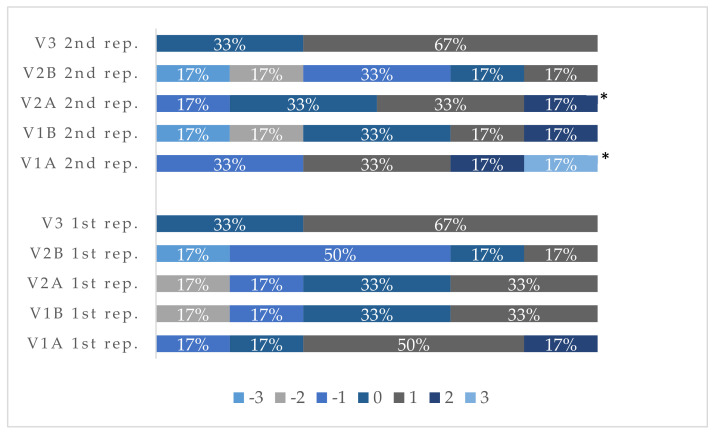
Frequency of subjective responses regarding thermal comfort. (V1A: +5 °C gloves A; V1B: +5 °C gloves B; V2A: −1 °C gloves A; V2B: −1 °C gloves B; and V3: +20 °C bare hands). (* *p* < 0.05).

At the end of each repetition, subjects answered questions about subjective thermal sensations, the results of which were shown in Figure 5.

Significant differences in subjective thermal sensations were observed between the variants in the 2nd repetition (*p* = 0.014). Subjective thermal sensations were lower when using gloves B compared to gloves A at both +5 °C and −1 °C conditions (V1A 2nd repetition and V1B 2nd repetition *p* = 0.043; V2A 2nd repetition and V2B 2nd repetition *p* = 0.043). In the case of gloves B and work at the temperature of −1 °C (V2B), the subjective thermal sensations of the participants were the lowest.

There were no differences between subjective ease of manipulation response or comfort of use in response between different variants (data not shown).

## 4. Discussion

This study showed that exposure to ambient temperature of 5 °C or lower, even when using protective gloves, lead to impaired ability to perform finger and hand dexterity tasks (manual dexterity), which was demonstrated by extending the time required to complete the specific tasks. In this study, manual performance was reduced within about 45 min of exposure to a cold environment, despite wearing gloves that meet certain standards for work in a cold microclimate. It follows that a relatively short exposure time in a cold environment can reduce manual dexterity, which is consistent with the research of other authors [5,18]. Traditional protective gloves are made from a combination of textile materials, sometimes with waterproof leather or coated materials. Protective glove materials should have a low heat transmission coefficient, while maintaining flexibility at low temperatures, which is required to perform manual tasks. In practice, increasing thermal insulation is achieved by using additional insulating inserts and linings or by double glove solutions [8,13]. Glove A and glove B tested in the presented study met the requirements of the protective parameters of thermal insulation and thermal resistance.

It is commonly believed that wearing gloves has a negative effect on manual dexterity (e.g., dexterity, grip strength) [3,10,11,12,18,23,30,33], but not all studies confirm this relationship [34]. Therefore, due to the influence of the type of gloves on dexterity, tests simulating manual tasks are important in the study of ergonomic properties of gloves [35]. One way to assess manual dexterity is to determine the time needed to complete a task. The type of gloves can influence on time required to complete the specific task [30,31]. Manual dexterity can also be influenced by the type of manual tasks related to the shape and type of surface of objects manipulated by workers [16,36,37]. However, the raw material as well as the micro- and macro-structure of the woven or knitted fabric have an important role in the construction and properties of the products [38].

In our study, the time to perform two repetitions of completed walls, as well as to perform single repetition using both types of gloves and at different temperatures, differed significantly from the time needed to complete the same task using only bare hands at a temperature of +20 °C. In other studies, it was also observed that manual performance was better at moderate temperatures [31]. However, it should be emphasized that, in contrast to our research, in the study of Geng et al. [31], the volunteers performed all tasks wearing gloves, even at a temperature of +19 °C. Differences in time required to complete the manual dexterity task were also observed in the study of volunteers without exposure to cold [30]. In the case of bare hands or cotton gloves, subjects completed the task faster than with nitrile or nylon gloves. The time needed to complete the task was 2%, 8% and 27% higher for cotton, nylon and nitrile gloves, respectively, compared to bare hands [30].

In our study, differences in the time required to complete the individual repetitions were also observed. Subjects completed the 2nd repetition faster than the 1st repetition of completion of all walls, when wearing double gloves (glove A) at +5 °C (00:11:15, 00:12:59, respectively). Moreover, less time was required to complete the wall 2.2 than wall 2.1, when subjects wearing glove B at 5 °C (00:04:38, 00:05:56, respectively). Dianat et al. [30] also observed that task completion times also differed between the beginning and the end of the task. During 90 and 120 min, participants took less time to complete the task than at the beginning (0 min) and at 30 min [30]. It is possible that although the participants were familiar with the manual tests in our study, the faster time to perform the same activity again is due to the learning effect.

Geng et al. [31] also noticed differences in the time needed to perform a bolt-nut task, but not for pick-up task at −10 °C, depending on the type of glove. They examined the wearing of single gloves or double gloves. In this case, when using double gloves and outer gloves, differences were observed for the pick-up task performance time, but not for the bolt-nut task [31]. In our work, it was also observed that the type of glove had an impact on the time needed to perform the task at a temperature of −1 °C. Using glove B (single glove), the time required to complete wall 3 was longer than using glove A (double glove) (00:02:16 and 00:01:47, respectively). These results were also confirmed in our study by the subjective responses concerning thermal sensation. Glove A was better rated by the subjects than glove B at both +5 °C and −1 °C, which is in line with the Geng et al. [31] study.

It would seem that wearing more than one glove should impair manual performance (hand dexterity) in cold environments; therefore, it is quite difficult to explain why some studies did not confirm this [31]. Geng et al. [31] suggest that: (1) the size of the glove was incorrectly matched to the hand, and the use of double gloves increased the fit; and (2) double gloves may increase the thermal insulation of gloves in a cold environment [31]. It is also possible that the single glove we used (glove B) was more massive/bulky and thicker, which may make it difficult to perform manual tasks (more hindrance to the manipulative tasks) because glove thickness can be one of the factors affecting dexterity [11,13,23,39]. Moreover, in the case of a single glove (glove B), greater thermal discomfort was observed than in the case of a double glove (glove A), which may suggest that the skin temperature of hands and fingers have been reduced to a threshold that impairs dexterity. It is suggested that finger skin temperature thresholds for finger dexterity/manual performance is about 14–15 °C [3,7,13,22,40,41]. It should also be noted that the palm part of the tested single glove (glove B) was made of polymer-coated textile materials. Other authors emphasized that in cold working environment the stiffness of such materials increases, which is unfavorable from the point of view of manual work. There is a reduction in the elasticity and a stiffening of the material [31,42].

It is difficult to compare the effects of gloves or ambient temperature on subjective sensation due to using different scales to assess perception by various authors. In our study, there were no differences between type of glove and rate of ease of manipulation (manual dexterity scale) as well as comfort of using of gloves between different thermal conditions. However, the type of glove may influence the subjective response of participants [30]. It was demonstrated that glove type affect rate of ease of manipulation as well as rate of hand and finger discomfort [30]. Subjective perception of ease of manipulation was between very easy and easy for bare hand, cotton and nylon gloves; this was in contrast to nitrile gloves for which the sensations were defined between easy and neutral [30]. Similarly, Basak et al. [9] described that subjective satisfaction levels and manual dexterity were affected by glove type, in that using the double glove obtained the lowest values for satisfaction levels and manual dexterity (5.34 for single glove, 5.20 for double glove, and 7.54 for powder and latex free glove; 9.02 for single glove, 4.20 for double glove, and 9.25 for powder and latex free glove, for satisfaction level and manual dexterity, respectively) [9]. Moreover, type of glove and hand skin temperature have an impact on discomfort ratings (determined by locally perceived discomforts in the hands) [11]. When hand skin temperature was 5 °C and 45 °C, the discomfort rating felt by the subjects increased compared to the hand skin temperature of 25 °C [11]. More discomfort was experienced by participants when they used neoprene, rubber and RY-WG002 gloves compared to bare-handed, cotton or anti-vibration gloves [11].

It should be noted that the study has limitations in terms of the number of gloves tested and the number of participants. The presented research is a pilot study and will be continued. Preliminary conclusions will provide a basis for further consideration, including the inclusion of a physiological factor in the studies. The basis of the presented work is the observations on the effect of temperature. In the presented study, it was observed that the time to complete wall 3.1 for glove A at +5 °C was longer than the time for glove A at −1 °C. Similarly, wall 3.2 also took longer in +5 °C than in −1 °C using glove A. This is probably due to a learning effect, even though the procedure was rotated, i.e., the test was started once with glove B, once with glove A and at different temperatures. It is also worth noting the physiological factor associated with the neuromuscular effect in cold working environment. This is associated with a slowing down of nerve conduction, which translates into an increase in muscle strength, and thus an increase in manual dexterity [43,44].

## 5. Conclusions

In our study, both the ambient temperature and the type of glove worn may influence the time required to complete a specific task, and thus affect the manual dexterity of workers. It was observed that time to complete the task was longer at temperatures of +5 °C than at −1 °C, which indicates the influence of temperature on manual dexterity. Moreover, time required to complete a task when wearing double gloves (glove A) was shorter than when wearing single gloves (glove B), which suggests the impact of glove type on manual dexterity. Therefore, it is important to choose gloves for workers that protect them as much as possible against the effects of cold air and mechanical and chemical injuries, while also limiting manual dexterity as little as possible [7]. Knowledge of the effects of type of glove on manual dexterity as well as on hand skin temperature can allow the employee to make the right decision when choosing the type of glove for the workplace, especially in extreme thermal condition environments [11,16].

## Figures and Tables

**Table 1:** Characteristics of tested materials for protective gloves A and B.

Glove A—Double Glove	Glove B—Single Glove
white glove: five-finger glove made of knitted, polyester fibres with increased mechanical parameters	a glove with a five-finger construction made of polyester fibres with an acrylic lining, with increased insulating properties; the outer side, dorsal and palm part of glove, was coated with polyacrylonitrile rubber with an uneven surface increasing grip in a wet environment
black glove: five-finger glove made of knitted fleece, polyester fibres with insulating properties	
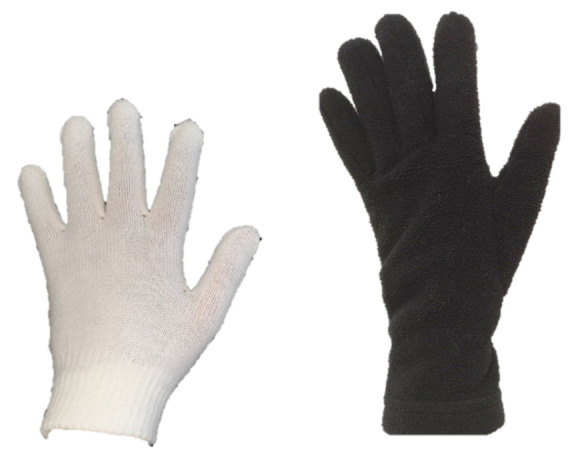	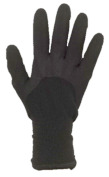

**Table 2:** Thermal comfort, easy of manipulation and comfort of using scales.

Thermal Comfort Sensation	Ease of Manipulation	Comfort of Use
−3 cold	1 making work impossible	1 very uncomfortable
−2 cool	2 interferes very much with work	2 uncomfortable
−1 slightly cool	3 moderately interferes with work	3 not very comfortable
0 neutral	4 little interferes with work	4 comfortable
1 slightly warm	5 does not interfere with work	5 very comfortable
2 warm		
3 hot		

**Table 3:** The results of thermal insulation (resistance to convective cold) of the protective gloves according to the standard EN 511, p. 5.5. [25].

Gloves	Thermal Insulation I_TR_ [m^2^ °C/W]	Mean Value of Thermal Insulation I_TR_ [m^2^ °C/W]	Performance Level	Compliance with the Requirements
Gloves A	0.098	0.10	1	Tested gloves meet the requirements of the standard—1st performance level
	0.098			
Gloves B	0.099	0.10	1	Tested gloves meet the requirements of the standard—1st performance level
	0.098			

**Table 4:** Results of thermal resistance (resistance to cold contact) of protective gloves according to the standard EN 511, p. 5.6. [25].

Gloves	Thermal Resistance R [m^2^ °C/W]	Mean Value of Thermal Resistance R [m^2^ °C/W]	Performance Level	Compliance with the Requirements
Gloves A	0.1010	0.097	2	The tested set meets the requirements standard—2nd performance level
	0.0938			
Gloves B	0.1030	0.102	3	The tested glove meets the requirements Standard—3rd performance level
	0.1000			

**Table 5:** Time (min) required to complete all walls in every variant of climate condition (mean ± standard deviation).

Variants	V1A	V1B	V2A	V2B	V3
Conditions	+5 °C, gloves A	+5 °C, gloves B	−1 °C, gloves A	−1 °C, gloves B	+20 °C, bare hands
Time of total task (min)	00:24:13 ± 00:01:49	00:24:48 ± 00:05:29	00:23:09 ± 00:04:24	00:25:00 ± 00:04:40	00:13:50 ± 00:01:25 *
Time of 1st repetition (min)	00:12:59 ± 00:01:04 **	00:12:51 ± 00:02:40	00:11:21 ± 00:02:08	00:12:29 ± 00:02:19	00:06:50 ± 00:00:57 *
Time of 2nd repetition (min)	00:11:14 ± 00:01:06	00:11:57 ± 00:02:55	00:11:48 ± 00:02:28	00:12:31 ± 00:02:29	00:06:59 ± 00:00:33 *

* variants V1A, V1B, V2A and V2B differed from the variant V3 (*p* < 0.05); ** V1A differed between 1st and 2nd repetitions (*p* < 0.05).

**Table 6:** Significant differences between variant V3 (control condition) and other variants (* *p* < 0.05). (V1A: +5 °C gloves A; V1B: +5 °C gloves B; V2A: −1 °C gloves A; V2B: −1 °C gloves B; V3: +20 °C bare hands; wall 1.1—wall 1, 1st repetition; wall 1.2—wall 1, 2nd repetition; wall 2.1—wall 2, 1st repetition; wall 2.2—wall 2, 2nd repetition; wall 3.1—wall 3, 1st repetition; wall 3.2—wall 3, 2nd repetition).

Variants	Time to Complete the Individual Walls (min)	V3 wall 1.1	V3 wall 2.1	V3 wall 3.1	V3 wall 1.2	V3 wall 2.2	V3 wall 3.2
Time to complete the individual walls (min)	Mean ± SD	00:02:35 ± 00:00:23	00:02:48 ± 00:00:17	00:01:27 ± 00:00:26	00:02:31 ± 00:00.13	00:02:54 ± 00:00:30	00:01:34 ± 00:00:11
V1A wall 1.1	00:05:23 ± 00:00:42	*	-	-	-	-	-
V1A wall 2.1	00:05:21 ± 00:01:21	-	*	-	-	-	-
V1A wall 3.1	00:02:15 ± 00:00:21	-	-	*	-	-	-
V1A wall 1.2	00:04:34 ± 00:00:46	-	-	-	*	-	-
V1A wall 2.2	00:04:28 ± 00:00:46	-	-	-	-	*	-
V1A wall 3.2	00:02:11 ± 00:00:19	-	-	-	-	-	*
V1B wall 1.1	00:05:37 ± 00:01:17	*	-	-	-	-	-
V1B wall 2.1	00:05:06 ± 00:01:33	-	*	-	-	-	-
V1B wall 3.1	00:02:08 ± 00:00:12	-	-	*	-	-	-
V1B wall 1.2	00:05:17 ± 00:01:53	-	-	-	*	-	-
V1B wall 2.2	00:04:38 ± 00:01:42	-	-	-	-	*	-
V1B wall 3.2	00:02:02 ± 00:00:24	-	-	-	-	-	*
V2A wall 1.1	00:05:01 ± 00:01:35	*	-	-	-	-	-
V2A wall 2.1	00:04:29 ± 00:00:46	-	*	-	-	-	-
V2A wall 3.1	00:01:54 ± 00:00:21	-	-	*-*	-	-	-
V2A wall 1.2	00:05:34 ± 00:01:36	-	-	-	*	-	-
V2A wall 2.2	00:04:27 ± 00:01:07	-	-	-	-	*	-
V2A wall 3.2	00:01:47 ± 00:00:17	-	-	-	-	-	-
V2B wall 1.1	00:05:56 ± 00:01:53	*	-	-	-	-	-
V2B wall 2.1	00:04:28 ± 00:00:20	-	*	-	-	-	-
V2B wall 3.1	00:02:05 ± 00:00:17	-	-	*	-	-	-
V2B wall 1.2	00:05:44 ± 00:01:37	-	-	-	*	-	-
V2B wall 2.2	00:04:31 ± 00:00:43	-	-	-	-	*	-
V2B wall 3.2	00:02:16 ± 00:00:28	-	-	-	-	-	*

## Data Availability

Not applicable.
